# Cytokines from the pig conceptus: roles in conceptus development in pigs

**DOI:** 10.1186/2049-1891-5-51

**Published:** 2014-11-07

**Authors:** Rodney D Geisert, Matthew C Lucy, Jeffrey J Whyte, Jason W Ross, Daniel J Mathew

**Affiliations:** Animal Sciences Research Center, University of Missouri, 920 East Campus Drive, Columbia, MO 65211 USA; Department of Animal Science, Iowa State University, 2356 Kildee Hall, Ames, IA 50011 USA

**Keywords:** Cytokines, Embryo development, Porcine conceptus, Pregnancy, Prostaglandins uterus

## Abstract

Establishment of pregnancy in pigs involves maintaining progesterone secretion from the corpora lutea in addition to regulating a sensitive interplay between the maternal immune system and attachment of the rapidly expanding trophoblast for nutrient absorption. The peri-implantation period of rapid trophoblastic elongation followed by attachment to the maternal uterine endometrium is critical for establishing a sufficient placental-uterine interface for subsequent nutrient transport for fetal survival to term, but is also marked by the required conceptus release of factors involved with stimulating uterine secretion of histotroph and modulation of the maternal immune system. Many endometrial genes activated by the conceptus secretory factors stimulate a tightly controlled proinflammatory response within the uterus. A number of the cytokines released by the elongating conceptuses stimulate inducible transcription factors such as nuclear factor kappa B (NFKB) potentially regulating the maternal uterine proinflammatory and immune response. This review will establish the current knowledge for the role of conceptus cytokine production and release in early development and establishment of pregnancy in the pig.

**Electronic supplementary material:**

The online version of this article (doi:10.1186/2049-1891-5-51) contains supplementary material, which is available to authorized users.

## Introduction

Establishment of pregnancy by the pre-implantation porcine conceptuses (embryo and extraembryonic membranes) requires extending the lifespan and progesterone secretion from the corpora lutea (CL) and appropriately contributing to the intricate interplay between the maternal immune system and attachment of the rapidly expanding trophoblast. Rapid (less than 1 h) elongation of the pig conceptuses across the uterine epithelial surface provides the physiological mechanism for the release of conceptus estrogens (maternal recognition of pregnancy signal) to rapidly redirect endometrial release of luteolytic prostaglandin F_2α_ away from endocrine movement (towards the uterine vasculature) to an exocrine secretion (into the uterine lumen) to enable CL maintenance. Porcine conceptuses are proteolytic and highly invasive outside the luminal environment of the uterus [1] but *in utero* the conceptuses are non-invasive (invasiveness controlled by the release of numerous endometrial protease inhibitors) resulting in the superficial epitheliolchorial type of placentation. The peri-implantation period of rapid trophoblastic elongation (Days 11 to 12) and attachment to the maternal uterine surface (Day 13 to 18) is essential for establishing sufficient placental uterine area for subsequent nutrient transport for piglet survival to term. Additionally, conceptus release of factors during this critical phase of pregnancy establishment also involves the stimulation of uterine secretion of histotroph and modulation of the maternal immune system. The semiallogeneic conceptuses ability to modify the maternal uterine environment into an environment favorable for growth and survival occurs through the activation of inducible transcription factors within the conceptus and uterine endometrium. Many genes activated by the conceptuses stimulate a tightly controlled proinflammatory response within the uterus [2–4]. A number of the cytokines released by the elongating conceptuses stimulate inducible transcription factors, such as nuclear factor kappa B (NFKB), which are thought to contribute to the maternal uterine proinflammatory and immune response [5]. Activation of NFKB is not limited to the immune system but can regulate cell differentiation, proliferation and survival. A number of recent reviews have described the complex nature for the role of growth factors and cytokines during implantation [5–9]. The following review will establish our current knowledge of the role of conceptus cytokine production and release in early development and establishment of pregnancy in the pig.

### Window of implantation

**Figure 1:**
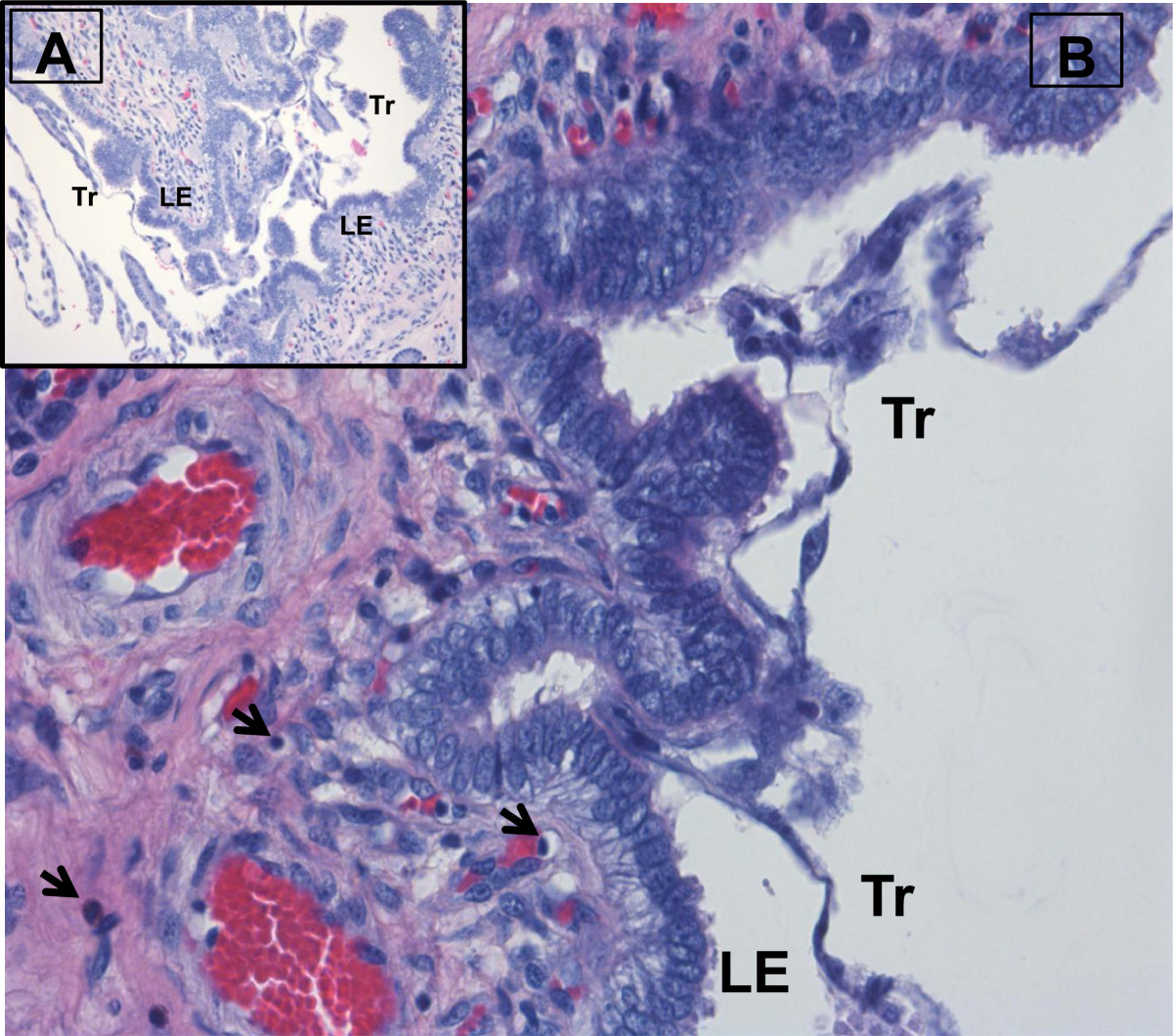
**Endometrial folding during pig conceptus attachment.** Following rapid trophoblast elongation on Day 12 of pregnancy, conceptus attachment to the endometrial surface epithelium induces a localized increase in endometrial surface folding on Day 14 of pregnancy **(A)**. Local conceptus release of IL1BE, IFN, estrogens or combination of the factors released by the conceptus to alter the uterine surface architecture (attachment and folding) to increase the surface area needed to support the epithiochorial type of placentation in the pig and alter immune cell trafficking to the uterine surface **(B)**. (Tr = trophectoderm, LE = luminal epithelium, arrows = lymphocytes in the underlying stratum compactum).

To fully appreciate the intricate interplay between the conceptus and uterus during the peri-implantation period requires a thorough understanding of the cellular localization and shifts in endometrial steroid receptors regulating the release of growth factors involved with conceptus development [4, 8]. Opening of the “window of receptivity” for trophoblastic elongation and attachment to the uterine luminal epithelium is regulated through ovarian estrogen and progesterone release and cell specific expression of steroid receptors within the uterine luminal (LE) and glandular (GE) epithelia and stroma. Although ovarian estrogen from the developing ovulatory follicles during proestrous and estrus is critical for priming the endometrium, progesterone and localization of its receptor play an essential role with cellular communication between the uterine epithelium and stroma in establishing a proper uterine environment for conceptus attachment and early development [10–12]. Progesterone’s role in opening the window for implantation during early pregnancy is associated with cell-specific changes in expression of endometrial progesterone receptor (PGR). Epithelial PGR (specifically PGRA) has been demonstrated to be a key regulator of uterine epithelial-stromal crosstalk essential for uterine development and function [13]. While uterine stromal and myometrial cells express PGR throughout pregnancy, a clear spatiotemporal association exists between the down-regulation of PGR in the endometrial LE and GE, and receptivity for conceptus implantation [11–16]. Down-regulation of PGR in endometrial epithelia is a conserved event among most mammals [14–20] and is associated with the down-regulation of high molecular weight mucin O-linked glycoproteins such as mucin 1 which serve as steric transmembrane inhibitors of trophoblast attachment [21–24]. A uterine environment permissive for peri-implantation conceptus development and activation of implantation is established through the loss of PGR from LE and GE cells. Maintenance of PGR in the stromal cell layer stimulates expression and secretion of progestamedins such as fibroblast growth factor 7 (FGF7) and hepatocyte growth factor [4, 10, 25] which in turn activate multiple uterine genes involved with growth, morphogenesis, synthesis of enzymes and enzyme inhibitors, extracellular matrix and cell adhesion prior to trophoblast attachment to the uterine surface [8, 12, 26, 27]. With cell specific loss of PGR from the LE and GE, estrogen receptor (specifically ESR1) is up-regulated in the uterine epithelium [28–30]. Establishment of a receptive endometrium for conceptus attachment is thus regulated through progesterone induction of epithelial PGR loss allowing finely synchronized alterations in the LE extracellular matrix exposing attachment factors such as transmembrane integrin heterodimer receptors and release of the matricellular protein, secreted phosphoprotein 1 (SPP1; also referred to as osteopontin) [3, 31] and balanced secretion of numerous growth factors, cytokines, prostaglandins, enzymes and their inhibitors which are enhanced by conceptus estrogen synthesis and release during the peri-implantation period [11, 27, 32]. Conceptus attachment and secretions also increase endometrial folding and LE proliferation (Figure 1) during early implantation in the pig [33]. The increase in endometrial folding and immune cell trafficking to the uterine surface may be induced by conceptus secretion of cytokines like interleukin 1β, interferons, estrogens or a combination of the conceptus release factors.

### Conceptus development

Opening the window of receptivity for conceptus attachment to the uterine endometrium (Day 10 to 14) following down-regulation of the uterine epithelial PGR marks a period of conceptus growth, development and change in morphology stimulated by the release of multiple uterine growth factors and cytokines [2–4]. During the early peri-implantation period, the endometrium increases the release of epidermal growth factor (EGF) [34–37], insulin-like growth factor-1 (IGF-1) [38–42], FGF7 [43, 44], vascular endothelial growth factor (VEGF) [45–47], interleukin 6 (IL6) [48–50], transforming growth factor beta (TGFB) [51–53], and leukemia inhibitory factor (LIF) [48–50] for which the developing conceptus trophectoderm expresses EGF-receptor (EGFR) [36], IGF1R [54], FGFR2 [55], VEGFR1 and 2 [45, 47], IL6R [50], TGFBR1 and 2 [52], and LIFR [50]. The increased endometrial release of EGF, FGF7, LIF, and IGF-1 are enhanced in the epithelium during the period of conceptus elongation and estrogen release [42, 44, 50, 51, 55]. Receptor activation by many of the uterine secreted factors has been shown to occur through multiple signaling pathways such as phosphatidylinositol 3-kinase (P13K)/AKT1 and mitogen-activated protein kinase ERK1/2MAPK [36, 47, 54] which are cell signaling pathways linked to stimulating trophectoderm proliferation, migration and survival. In addition to stimulating proliferation of trophoblast cells, TGFB, LIF and IL6 increase cell viability and attachment *in vitro*[50–52].

Growth of the early developing porcine conceptuses stimulated through the release of uterine growth factors is essential for achieving a critical developmental threshold that triggers rapid trophoblast expansion within the uterine lumen. Timing for the increased release of growth factors is dependent upon the length of progesterone stimulation which facilitates down-regulation of epithelial PGR in the endometrium [2, 3]. Several studies have elegantly demonstrated the impact of the duration of progesterone priming in that exogenous progesterone immediately following ovulation accelerates early conceptus growth in both sheep [56, 57] and cattle [58–60]. Administration of progesterone shortly after ovulation advances down-regulation of epithelial PGR by two days during the normal estrous cycle and pregnancy [56–60]. The advancement of epithelial PGR down regulation accelerates the release of uterine growth factors for the developing sheep conceptus [61].

**Figure 2:**
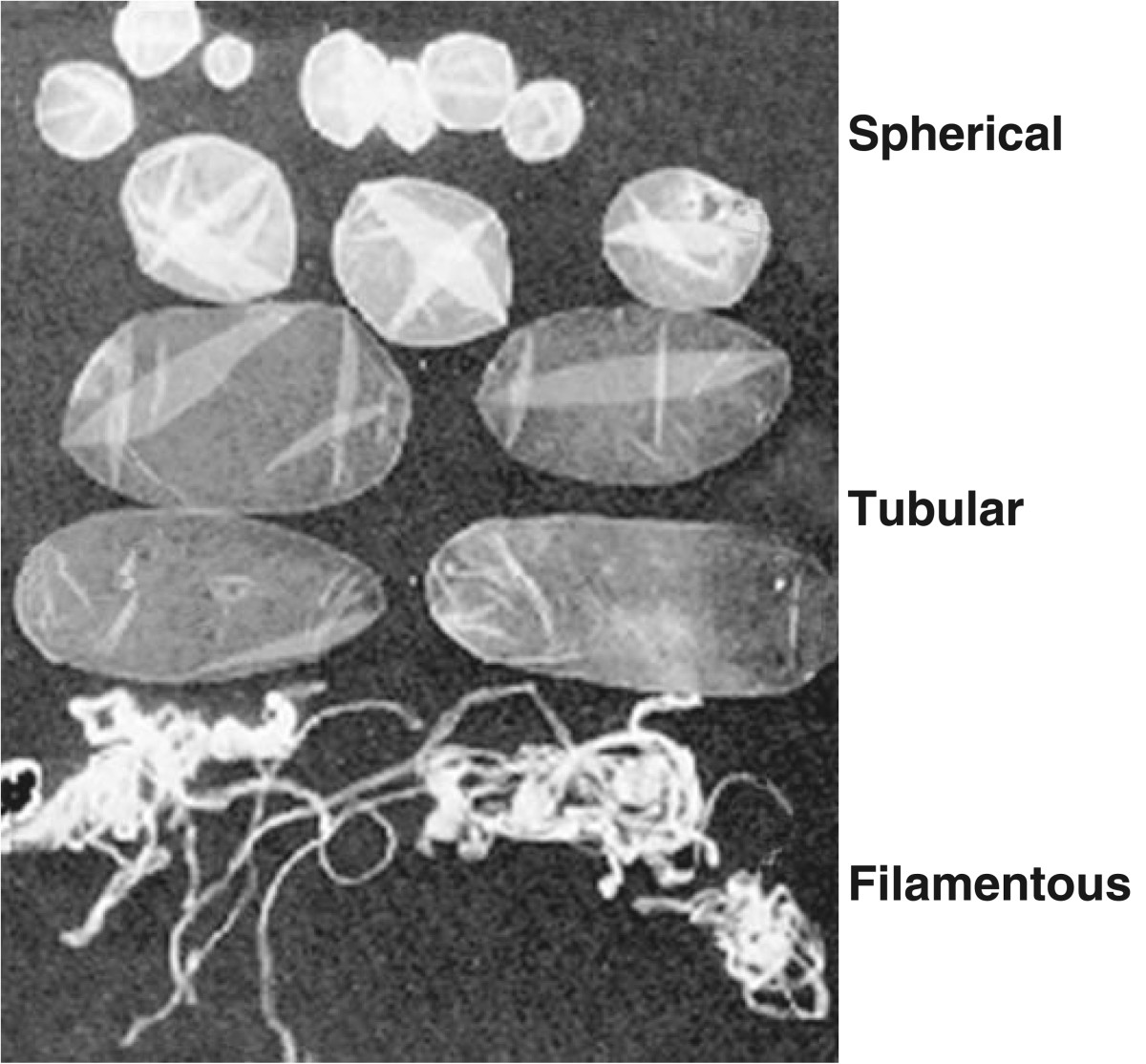
**Morphological stages of early conceptus development between Days 10 to 12 of pregnancy.** Upon reaching a spherical diameter of appropriately 10 mm, conceptuses rapidly transition to ovoid, tubular and filamentous morphologies within 2 to 3 hours.

Release of the uterine growth factors is clearly involved with growth and differentiation of the porcine conceptuses following hatching from the zona pellucida on Days 6–7 of gestation. Following hatching, peri-implantation development in the pig is unique in that conceptuses develop from a 1–2 mm sphere to a 9–10 mm long ovoid shape between Days 10 to 12 of pregnancy and then rapidly transition to tubular and filamentous forms by elongating at 30–40 mm/h to >100 mm in length (Figure 2) in 1 to 2 h [12, 33, 62]. Rapid conceptus elongation provides the mechanism for delivery of estrogen across the uterine surface to maintain CL function, stimulate secretions from the uterine LE and GE which are closely linked to initiation of trophoblast attachment to the uterine LE and establish individual placental surface area for nutrient absorption from the underlying endometrium for individual conceptuses [3, 23, 63].

The specific factor(s) involved with triggering the rapid morphological transformation of the ovoid conceptus to its filamentous shape is currently unknown. Although endometrial release of growth factors is involved with conceptus growth and development, variation in stages of development prior to and during the time of trophoblast elongation (spherical, ovoid, tubular and filamentous conceptuses present within the same litter) indicate that elongation is not necessarily triggered by a uterine-stimulated event but rather a specific stage of conceptus differentiation and development [33, 62, 64–68]. Rapid conceptus elongation does not occur through cellular hyperplasia but rather cellular remodeling [62]. The morphological alteration in shape of the trophectoderm and transformation of the underlying endoderm forming filapodia provides a mechanism to physically move cells into the elongation zone [62]. The focal point for the cellular restructuring occurs from the ends of the epiblast forming an extended band of cells (elongation zone) to the elongating tips of the conceptus trophectoderm [3, 62]. The force necessary for the cellular restructuring of the trophoblast during elongation occurs through modifications in microfilaments and junctional complexes [3, 62, 69, 70]. Elongation of the conceptuses may involve interaction of integrins on the endometrial LE apical surface [71].

As previously stated, timing of rapid conceptus elongation is established by the conceptus achieving a specific stage of development which is temporally associated with gastrulation and formation of the extraembryonic mesoderm [65, 72–74]. Yelich et al. [72] first indicated that 6 mm spherical conceptuses expressed gene transcripts for brachyury (marker for mesoderm formation) which precedes the initial detection of mesodermal outgrowth in 10 mm ovoid conceptuses. The increase in brachyury expression is associated with an alteration in steroidogenesis in the developing conceptuses [75]. Valdez Magana et al. [68] recently reported that epiblast development and differentiation provides the paracrine signaling between the epiblast and trophectoderm for trophoblast proliferation and mesoderm differentiation. Transcripts for FGF4 are highly detectable in the porcine epiblast but absent/low in the trophectoderm [68, 76]. However in ovoid conceptuses, FGFR2 is expressed in trophectoderm cells where there is abundance of FGF4 ligand which activates MAPK phosphorylation [68]. In addition, bone morphogenetic protein 4 (BMP4) expression in the developing extraembryonic mesoderm outgrowth from the epiblast that occurs between trophectoderm and endoderm stimulates BMPR2 in trophectoderm (absent in epiblast and hypoblast). Valdez Magaña et al. [68] suggested that increased epiblast production of FGF4 and expression of FGFR2 in the adjacent trophectoderm cells trigger the signaling cascade for trophoblast elongation. The novel suggestion that FGF4 is involved in the initial response of the conceptus is supported by information which indicates that FGF4 is not normally released into the extracellular fluid but moves in a gradient only over a short distance of a few cells [77, 78]. Induction of FGF4 in the epiblast stimulating MAPK in the trophectoderm through FGFR2 could coordinate with the extraembryonic mesoderm production of BMP4 to initiate the cascade of events involved with modifying microfilaments and junctional complexes necessary for the elongation process.

Although formation of the extraembryonic mesoderm in the conceptus is clearly a marker for the time of rapid trophoblast elongation and the cellular alternations involved, the conceptus factor triggering elongation of the porcine conceptus is unknown. Although conceptus elongation has not been achieved *in vitro*, it is clear that the conceptus activates elongation at a specific stage of development. Presence of spherical with filamentous conceptuses within the same litter [12] and the failure to advance elongation in vivo through estrogen administration prior to a stage of development for elongation [62, 67] demonstrate that initiation of trophoblast elongation is regulated by conceptus development. However, alterations in uterine secretion do have a direct impact on the rate of conceptus development to reach the stage for elongation.

A number of studies have evaluated the transcriptome of developing spherical, ovoid, tubular and filamentous pig conceptuses prior to and during elongation [64–66, 72, 79, 80]. These studies described a multitude of transcripts involved with steroidogenesis, lipid metabolism, cell morphogenesis, calcium binding, protein binding and nucleotide binding. Specific transcripts involved in steroidogenesis, such as steroidogeneic acute regulatory protein, cytochrome P450 side chain cleavage protein, 17α-hydrolase and aromatase all increase in abundance as the pig conceptuses approach and initiate the elongation process [64, 65, 72]. However, although administration of estrogen can advance uterine gene expression and secretions associated with the increase in conceptus estrogen production at elongation; it does not induce premature elongation of the conceptuses [81]. A number of transcripts involved with embryonic development, attachment and immune cell regulation such as s-adenosylhomocysteine hydrolase [79], retinoic acid receptors and retinol binding protein [72], TGFB [64, 72], LIFR [72], interferon-γ (IFNγ), B-cell linker, and chemokine ligand 14 [66] are altered during early conceptus development. The most striking change in the conceptus transcriptome during the transition from ovoid to filamentous morphology is the increase in expression of interleukin 1β (*IL1B*) [79, 80]. The increase in *IL1B* during transition to the filamentous form of porcine conceptus development was first described by Tuo et al. [82]. Interleukin 1β is a proinflammatory cytokine which is dependent on the expression of members of the IL-1 system belonging to the IL1B/Toll-like receptor (TLR) superfamily. The IL-1 system consists of two agonists (IL1A and IL1B), two receptors (IL1R1 (functional) and IL1R2 (pseudo-receptor)), converting enzymes, a receptor accessory protein (IL1RAP), and multiple isoforms of receptor antagonists (IL1Rant) [5, 83] which are all present in the porcine endometrium and conceptuses [79, 84, 85].

### Conceptus IL-1β

Conceptus *IL1B2* mRNA abundance rapidly increases during trophoblast elongation, but decreases over 2000-fold immediately following completion of the elongation process [86]. Based on the timing and pattern of conceptus IL1B release and the presence of the IL-1 system in the conceptuses and endometrium, Ross et al. [86] proposed that conceptus IL1B secretion was the signal to initiate the cascade of events involved with the rapid elongation process.

Recently, analyses of pig genome sequences and expressed sequence tags (EST) indicate that gene duplication resulted in two *IL1B* genes on *Sus scrofa* chromosome 3. The classical *IL1B1* is expressed in macrophages and endometrial tissue while the embryonic form (*IL1B2*) is only detected in the early porcine conceptus prior to attachment to the uterine LE [2, 87]. *IL1B2* is considered novel because the sequence is not expressed in other mammals [88]. The two predicted protein sequences are 85% identical and are least homologous near the N-terminus as caspase-1 cleaves this portion of the peptide resulting in a functional protein (D.J. Mathew, M.C. Lucy and R.D. Geisert unpublished results). Interestingly, in the embryonic form there is a proline inserted 2 amino acids from the predicted caspase-1 cleavage site. While the two genes are very similar from exon 2 to exon 7, exon 1 and the active promoter regions are different between the two genes. The promoter differences may partially explain variation in mRNA expression between the two forms. Activity and cell specificity of the two forms may also differ as recombinant IL1B2 can activate NFKB in alveolar macrophages and uterine surface epithelium but has reduced activity compared to recombinant IL1B1 (D.J. Mathew, R.D. Geisert and M.C. Lucy unpublished results).

Porcine IL1B2 is secreted only within a brief window associated with the morphological and functional changes that take place in conceptus development and elongation on Days 10 to 12 of pregnancy [86]. It has been postulated that one function of IL1B2 is to act as an inflammatory mediator in the endometrium [89]. Following synthesis and secretion by the conceptus, IL1B2 may trigger a cascade of signaling events that activate the transcription factor, NFKB in the LE of the endometrium. NFKB activation is an important component in opening the implantation window in pigs and other mammals [90]. Genes transcriptionally regulated by NFKB are involved in inflammation, immune function, cell adhesion, and release of cytokines, growth factors, anti-apoptotic factors and immunoreceptors [91]. The activation of inflammatory pathways in the endometrium likely enhances progesterone-induced uterine receptivity for conceptus implantation. It is important, however, that the inflammation cascade triggered by IL1B2 be tightly regulated in order to prevent rejection of the semi-allogeneic conceptus [9]. Conceptus estrogen release during elongation may play a key role in counter-balancing the increased inflammatory response by activating estrogen receptor (ESR1) which can affect the transcriptional activity of NFKB [90]. Thus, conceptus expression of IL1B2 would be consistent with the continued activation of NFKB, whereas the synchronous estrogen secretion by pig conceptuses may pose a suppressive effect to prevent an inflammatory reaction that would be detrimental to conceptus survival [2]. Interleukin-1β increases aromatase expression within human cytotrophoblast [92] and the increased synthesis of IL1B2 by pig conceptuses is temporally associated with elevated conceptus aromatase expression and the acute release of estrogen into the uterine lumen [72, 86]. Thus the increase in expression of both IL1B2 and estrogen by individual conceptuses that are expanding through the uterine lumen would counter-balance stimulation of the pro-inflammatory and immune response within the uterus.

IL1B2 may have other roles in rapid conceptus elongation and the regulation of maternal recognition. IL1B is an inducer of phospholipase A2 [93] and thus up-regulates cell membrane arachidonic acid release, thereby increasing membrane fluidity that is necessary for remodeling of the trophectoderm during elongation [2, 94]. The arachidonic acid could also be converted to prostaglandins which are needed for placental attachment during the establishment of pregnancy. Recent results from studies with ewes suggest that IL1B could play a role in regulating prostaglandin-endoperoxide synthase 2 (PTGS2) and the subsequent synthesis of prostaglandins that control conceptus elongation [95]. Pig conceptus IL1B2 secretion, therefore, may be of pivotal importance in the rapid morphological transformation of the pig conceptuses on Day 12 of pregnancy.

IL1B2 activation of NFKB stimulates prostaglandin synthesis through induction of PTGS2. IL1B1 increases endometrial IL1R1 and in conjunction with estrogen, IL1RAP, suggesting that IL1B2 and estrogen regulate endometrial transcriptional activity of NFKB during elongation [85, 86, 96]. IL1B has a stimulatory effect on endometrial prostaglandin E_2_ (PGE_2_) secretion and PTGS1 and PTGS2 mRNA expression from Days 10 to 13 of pregnancy [85, 97–99]. The presence of PGE_2_ receptors in the CL and endometrium [98] suggests that conceptus PGE_2_ secretion could also affect maintenance of the CL and directly stimulate adhesion and attachment of the trophoblast to the uterine epithelium [100]. Conceptus secretion of IL1B2 into the uterine lumen may also enhance endometrial expression of LIF and IL6 [50] possibly through activation of NFKB within the uterine LE and GE. IL1B1 induces human endometrial expression of LIF [101–103] and IL6 in placental villous core mesenchymal cells *in vitro*[104]. Suppression of NFKB activity in the endometrium alters the timing of implantation in the mouse which can be partially rescued by LIF supplementation [105]. LIF and IL1B stimulate expression of fucosyltransferase enzymes which are involved with embryo attachment to the uterine surface epithelium in the mouse [106]. During and following rapid conceptus elongation in the pig, there is increased endometrial secretion of LIF and IL6 [48–50]. Both *LIFR* and *IL6R* mRNA are detected in porcine conceptus [49, 50] suggesting that endometrial secretion of LIF and IL6 may play an important role in conceptus development and attachment to the uterine surface. Blitek et al. [50] indicated that LIF and IL6 stimulated proliferation and attachment of porcine trophoblast cells *in vitro*. Conceptus estrogen and IL1B2 secretion serve as major components in the embryo-uterine crosstalk to stimulate endometrial LIF and IL6 to contribute to the pathway for conceptus attachment to the uterine luminal surface.

Several papers have investigated endometrial differential gene expression between cyclic and pregnant pigs which provide numerous endometrial genes and pathways that the conceptus stimulates during the period of conceptus elongation and attachment [107–110] which will not be covered in this review. One interesting gene differentially expressed during pregnancy is *IL11RA*[110]. IL11 and its receptor (IL11RA) is proposed to prevent the invasion of trophoblast cells in the mouse [111] and human [112]. Although gene expression IL-11RA is lower in endometria of pregnant pigs, there was a pregnancy-specific increase in IL11RA on the surface epithelium [110]. As previous indicated porcine conceptuses are proteolytic and highly invasive outside the luminal environment of the uterus [1]. Therefore in addition to endometrial release of protease inhibitors during trophoblast attachment, porcine endometrial expression of IL11RA may serve to help inhibit the proteolytic trophoblast invasion through the surface epithelium during attachment [110].

### Switch to endometrial IL-18

Porcine conceptus *IL1B2* gene expression and secretion is clearly temporally associated with the rapid conceptus elongation as a dramatic reduction in mRNA abundance is soon followed by a depletion of IL1B2 protein in the uterine lumen following conceptus elongation on Day 12 [86]. The loss of conceptus IL1B2 secretion following elongation suggests that another closely related cytokine may function at the conceptus-uterine interface to continue regulation of the immunological interactions necessary for establishment of pregnancy in the pig. Interleukin 18 (IL18), also referred to as interferon-γ inducing factor [113], is a member of the IL-1 family of pro-inflammatory cytokines believed to play a significant role in implantation. Following the loss of conceptus IL1B2 stimulation, there is a switch to endometrial IL18 production and release during placental attachment in the pig [114]. Porcine endometrial *IL18* mRNA expression increases from Days 10 to 15 of the estrous cycle with mRNA expression increasing 10-fold on Day 18 of pregnancy. However, there is a pregnancy-specific increase in uterine luminal content of IL18 between Days 15 and 18 due to an increase in caspase-1 expression induced by the developing conceptuses [114]. Caspase-1 cleaves and activates the proforms of both IL1B and IL18 [115]. Pro-IL18, which has structural similarities to pro-IL1B, is involved with modulation of the immune system through induction of interferon-γ [116]. Conceptus secretion of IFNG increases immediately following trophoblast elongation in the pig [117], suggesting that the conceptuses may induce endometrial IL18 release to assist in development and placental attachment during early pregnancy. Interestingly, unlike IL1B which is stimulated by inflammatory responses in cells, IL18 is stored in healthy cells and its biological activity is dependent upon its release through caspase-1 processing [118]. Although similar to IL1B, IL18 binds to a unique IL18 receptor which consists of two receptor chains, ligand-binding chain IL18RA and a co-receptor IL18RB chain (similar to IL1B accessory protein), which are required for cellular signaling [119]. The conceptus factor that stimulates the increase in caspase-1 in the uterine epithelium is unknown, although IL1B2 could stimulate release IL18 from the uterine epithelial cells through increasing caspase-1 activity [120]. Biological activity of IL18 is regulated through release of an IL18 binding protein (IL18BP) which functions as a negative feedback loop to suppress IFNG production and limit Th1 cell responses.

The increased endometrial expression of caspase-1, and release of IL18 into the uterine lumen may stimulate expression and secretion of IFNG by conceptuses [117] to modulate the maternal immune system through signal transducer and activator of transcription 1 (STAT1) at the interface between trophectoderm and uterine LE [121]. The loss of conceptus IL1B2 stimulation and switch to endometrial IL18 production during placental attachment in the pig would decrease the potential pro-inflammatory stimulation of the conceptuses following trophoblast elongation which maybe important to control cytokine and immune functions following implantation [122]. Increased secretion of IL18 at the uterine/trophoblast interface is associated with increased pregnancy rates in one line of abortion-prone mice [123].

### Conceptus interferons (IFN)

During the peri-implantation period of conceptus attachment to the uterine LE following trophoblastic elongation, pig conceptuses secrete of IFNG (Type II IFN) and IFND (Type I IFN) between Days 12 to 20 of gestation [117, 121, 124]. Trophoblastic production and secretion of two IFNs, of which IFNG is the predominate form [125, 126], is unique compared with other mammalian species. Trophoblast secretion of IFNG and IFND would enable activation of a distinct gene set through two different receptors that may provide a uniquely regulated stimulation within the endometrium [127]. With the abrupt decline in conceptus expression of IL1B2 following rapid elongation, there is a tremendous increase in the filamentous conceptus trophoblastic expression of specifically IFNG during initiation of attachment to the uterine LE on Day 13 [66, 117, 121]. Unlike IFNT produced by the conceptus of ruminant species, pig trophoblastic IFNs do not directly function as a maternal recognition signal for CL maintenance [3]. However, pig IFNG and IFND can increase endometrial PGE_2_ secretion [128] and induce cell-specific endometrial IFN-stimulated genes [127, 129].

Joyce et al. [121] suggested that conceptus estrogens and IFNs regulate endometrial IFN-stimulated genes through a cell-type-specific manner. Conceptus secretion of estrogen increases STAT1 in LE to initiate the signal for pregnancy recognition and CL maintenance as well as inducing changes to the apical surface glycocalyx of LE to allow conceptus attachment. Conceptus IFNG and IFND induced increases of STAT1 are limited to the underlying endometrial stromal cells that express interferon regulatory factor 1, IFNG/STAT1-responsive gene, that is absent in the LE [121]. Pig conceptuses secrete estrogen during the peri-implantation period of pregnancy which increases uterine LE expression of interferon regulatory factor 2 (IRF2), a transcriptional repressor of classical IFN-stimulated genes, which would also restrict IFNG and IFND stimulation to the underlying stroma. Thus expression of classical IFN responsive genes such as MX1, interferon stimulated gene 15 (ISG15), IRF1, STAT1 and STAT2 are localized in the stroma and GE in pigs [121]. The cell specific activation by the pig trophoblastic IFNs may play an essential role in regulating the immunological barrier for attachment of the semi-allogeneic conceptuses [3, 130]. MHC class I molecules such as SLA and β2-microgobulin which are involved with recognition of foreign cells and pathogens are not expressed on the trophoblast and are absent in early pregnancy of the pig [127]. The increase in uterine angiogenesis which occurs during the peri-implantation period between Days 13 to18 of pregnancy [131] could also be stimulated through the trophoblast secretion of IFNs in addition to other conceptus and uterine angiogenic factors such as VEGF.

## Conclusion

**Figure 3:**
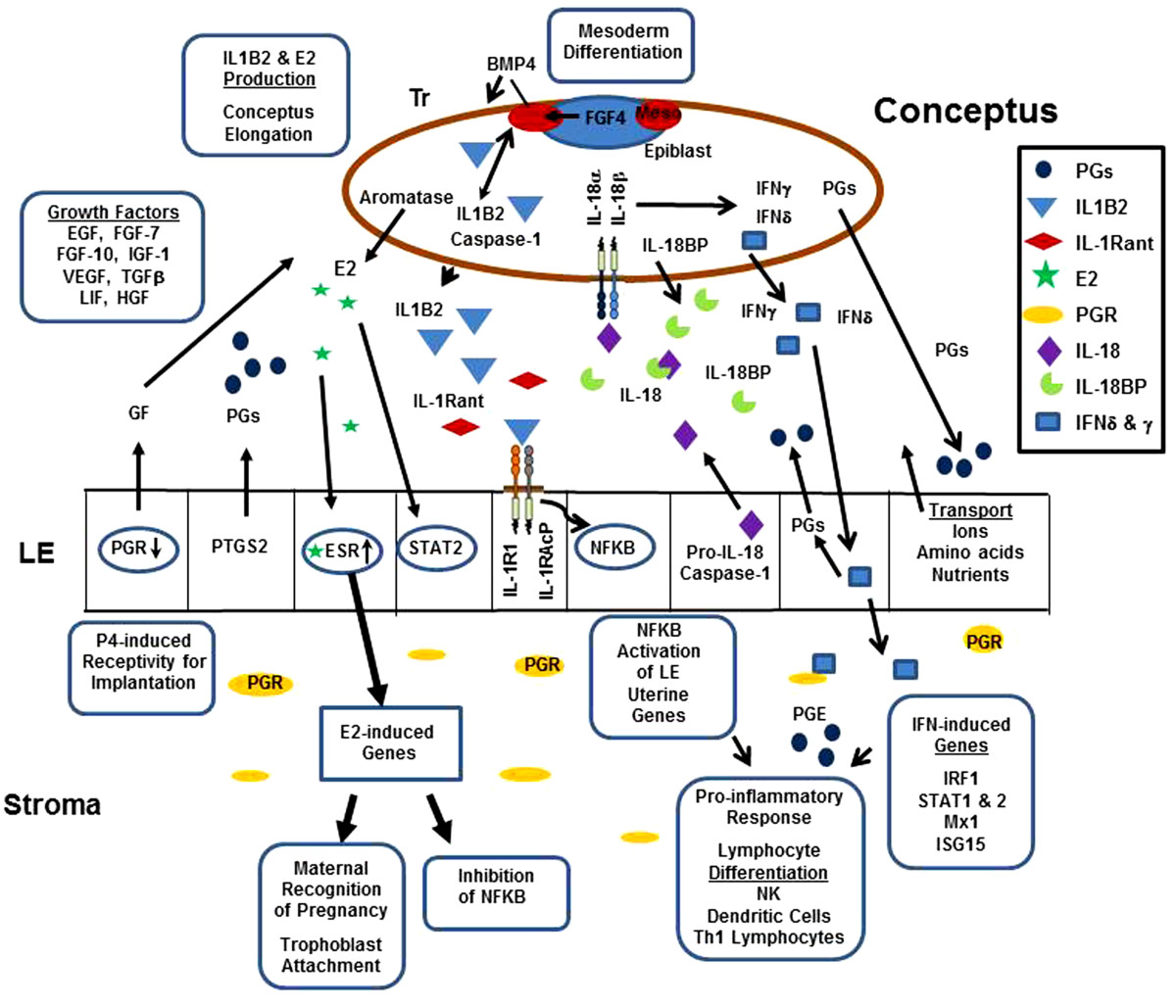
**Summary of conceptus/uterine interactions from Day 12 to 18 of pregnancy.** Exposure of the endometrium to progesterone secretion induces down-regulation of progesterone receptor (PGR) in the endometrial surface (LE) and glandular epithelium (GE). Progesterone modulation of uterine function is maintained by the presence of PR in stromal cells. Down-regulation of PGR in LE opens the window of receptivity of conceptus attachment to the endometrial surface. Progesterone stimulation increases PTGS2 within the LE increasing release of PGF2α into the uterine vasculature inducing CL regression during the estrous cycle. On Day 11 to 12 of pregnancy, conceptus epiblast expression of FGF4 stimulates production of BMP4 by the trophectoderm (Tr) to trigger differentiation of the mesoderm (meso) which may lead to induction of pathways to trigger conceptus trophoblast elongation. Embryonic IL1B2 initiates cellular remodeling during elongation and activates NFKB in the LE through binding to a functional IL1 receptor (IL1RI) and its receptor accessory protein (IL1RAcP). Activation of NFKB induces endometrial genes involved with inducing a pro-inflammatory response. IL1B2 activity in the conceptus and uterus is regulated through the level of receptor antagonist (IL1Rant) expression. Conceptus aromatase expression enhances estrogen secretion, which binds to ESR in the LE and GE increasing endometrial PGE production and altering the movement of PGs into the uterine lumen, thereby preventing luteolysis and maintaining pregnancy. Estrogen induction of STAT2 stimulates endometrial changes needed for placental attachment and may also play a role in modulating NFKB pro-inflammatory responses. Following conceptus elongation, IL1B2 expression ceases but is immediately replaced by expression of IFNγ and IFNδ and increased release of IL-18 into the uterine lumen. The activity of IL-18 is regulated through the concentration of its binding protein (IL-18BP). Activation of IFN-induced genes and conceptus PGE production may help regulate the pro-inflammatory response and regulate lymphocyte differentiation and activation within the uterine stroma and epithelium.

Proper timing for conceptus growth and development is proposed to be regulated through the initial down-regulation of PR in the uterine LE which stimulates growth factors to promote mesodermal differentiation and expression of FGF4 and BMP4 that initiate conceptus IL1B2 expression and release to stimulate rapid elongation of the conceptuses throughout the uterine lumen (Figure 3). Expansion of the conceptuses throughout the uterine horns provides the mechanism for estrogen to cover the uterine surface for maternal recognition of pregnancy, initiate trophoblast attachment to the LE and regulate the maternal lymphocyte response to conceptus IFNs which stimulate vascular changes and increases angiogenesis for the proper microenvironment for placentation.

The role of IL18 and IFNG in regulating Th1 lymphocytes and natural killer (NK) cell responses in tissues suggests that pig conceptus secretion of estrogens, IL1B2, prostaglandins, IFNs and endometrial release of IL18 serve to not only induce cell surface adhesion factors for trophoblast attachment, but also play a critical role in balancing the immune cell migration and recognition of receptors to support or reject the developing embryos and their extraembryonic membranes. The IL-1 family of cytokines plays a critical role in the regulation of immune cell differentiation and activity during pregnancy as well as many inflammatory diseases [132]. During pregnancy in the pig, the conceptus recruits uterine natural killer lymphocytes, dendritic cells and other immune cells at the sites of trophoblast attachment which induce major changes in the endometrial vasculature and angiogenesis to support the developing conceptus [133, 134]. Although not demonstrated in the uterus of the pig, the increase in PGE_2_ from the conceptuses and endometrium may play a role in minimizing pro-inflammatory tissue damage through switching from leukotriene B_2_ synthesis to lipoxin A_4_ and release of the anti-inflammatory resolvins and protectins [135]. Clearly pig conceptuses release a number of paracrine factors at the maternal/placental interface to regulate the vascular, angiogenic and immune changes needed to establish pregnancy (Figure 3). The conceptus IL-1 family of cytokines is but one component of a larger group of signaling pathways involved with successful survival of developing embryos. However, pregnancy is not only dependent upon the presence of the various cytokines during implantation but also in the appropriate timing of their release.

It is well established that exposure of pregnant gilts to exogenous estrogen 48 h prior to normal conceptus release at elongation on Day 12 results in fragmentation of the conceptus between Days 15 to 18 of pregnancy [136, 137]. Premature exposure of the endometrium to estrogen advances expression of multiple genes during the period of trophoblast elongation and attachment [138]. Most of the aberrantly expressed endometrial genes are those involved with immune cell regulation and cell adhesion. Early estrogen exposure (Days 9 and 10) of pregnant gilts does not affect endometrial *IL18* mRNA expression but disrupts the normal LE release of IL18 into the uterine lumen [114]. Although caspase-1 increases between Days 12 to 18 in estrogen-treated gilts, there is no increase in the luminal content of IL18 as occurs in untreated pregnant gilts. Lack of IL18 release from the LE may directly affect conceptus expression of IFNG. Although STAT1 expression is present in the LE, stromal expression of STAT1 is absent in estrogen-treated gilts [121]. These data indicate a temporally regulated presence of intricate interactions between conceptus estrogen, IL1B2, IFNG and uterine IL18 release in programing downstream transcription factors needed to establish pregnancy in the pig.

## Authors’ information

RDG is a Reproductive Physiologist in Division of Animal Science at the University of Missouri, Columbia where his research program over the past 30 years has investigated the interaction between the early developing porcine conceptuses and uterus. MCL is a Reproductive Physiologist in Division of Animal Science at the University of Missouri, Columbia where his research program is focused on dairy cattle reproduction and estrous synchronization. DJM is currently a doctoral graduate student completing his research program on porcine conceptus elongation and establishment of pregnancy in the pig. JWR is a Reproductive Physiologist in the Department of Animal Science at Iowa State University, Ames where he has established a research program on small RNA regulation of reproductive function and the effects of heats stress on pig development. JW is a Reproductive Physiologist in Division of Animal Science at the University of Missouri, Columbia where his research program is involved with the development of transgenic pigs for research in development and disease models.

## Authors’ original submitted files for images

Below are the links to the authors’ original submitted files for images. Authors’ original file for figure 1 Authors’ original file for figure 2 Authors’ original file for figure 3

## Abbreviations

BMP4: Bone morphogenic protein 4
BMPR2: Bone morphogenic protein receptor 2
CL: Corpora lutea
EGE: Epidermal growth factor
EGFR: Epidermal growth factor receptor
ESR: Estrogen receptor
EST: Expressed sequence tags
FGF: Fibroblast growth factor
FGFR2: Fibroblast growth factor 2 receptor
GE: Glandular epithelium
IGF: Insulin-like growth factor
IGF-1R: Insulin-like growth factor 1 receptor
IFN: Interferon
IRF: Interferon regulatory factor
IL: Interleukin
IL-6R: Interleukin 6 receptor
IL-1β2: Interleukin 1β conceptus form
IL-1RAP: Interleukin 1 receptor accessory protein
IL-1Rant: Interleukin 1receptor antagonist
IL-1RT1: Interleukin 1 receptor type 1
LIF: Leukemia inhibitory factor
LIFR: Leukemia inhibitory factor receptor
LE: Luminal epithelium
Mx1: Interferon-induced GTP-binding protein
NFKB: Nuclear factor κB
PR: Progesterone receptor
PG: Prostaglandin
PTGS2: Prostaglandin endoperoxide synthase 2
STAT: Signal transducer and activator of transcript
TGFβ: Transforming growth factor beta
TGFBR: Transforming growth factor beta receptor 1
VEGF: Vascular endothelial growth factor
VEGFR: Vascular endothelial growth factor receptor
