# Supplementary figures and images for: Cytokines from the pig conceptus: roles in conceptus development in pigs
Source: J Anim Sci Biotechnol. 2014 Nov 7;5:51. doi: 10.1186/2049-1891-5-51 (PMC4247618; doi:10.1186/2049-1891-5-51)

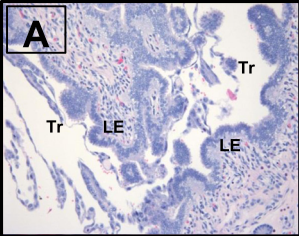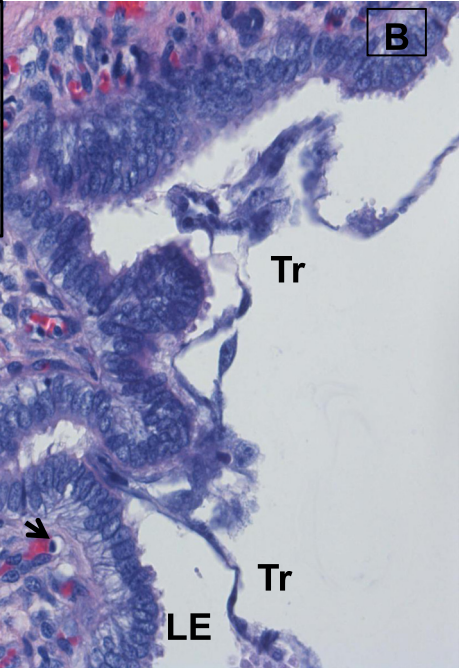

Supplement: Supplementary file 1 — Authors’ original file for figure 1 [file 40104_2014_129_MOESM1_ESM.pdf]

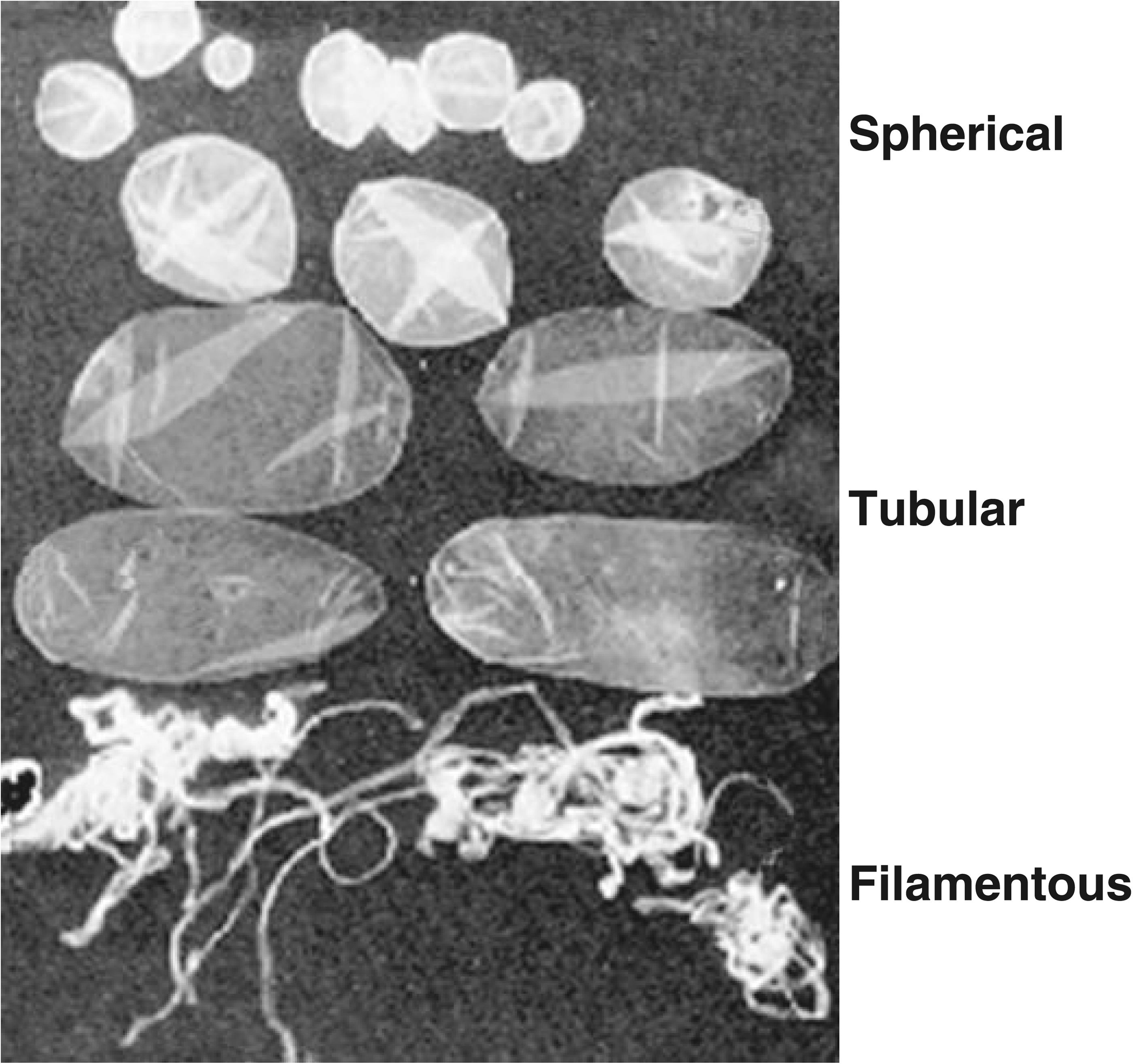

Supplement: Supplementary file 2 — Authors’ original file for figure 2 [file 40104_2014_129_MOESM2_ESM.tif]

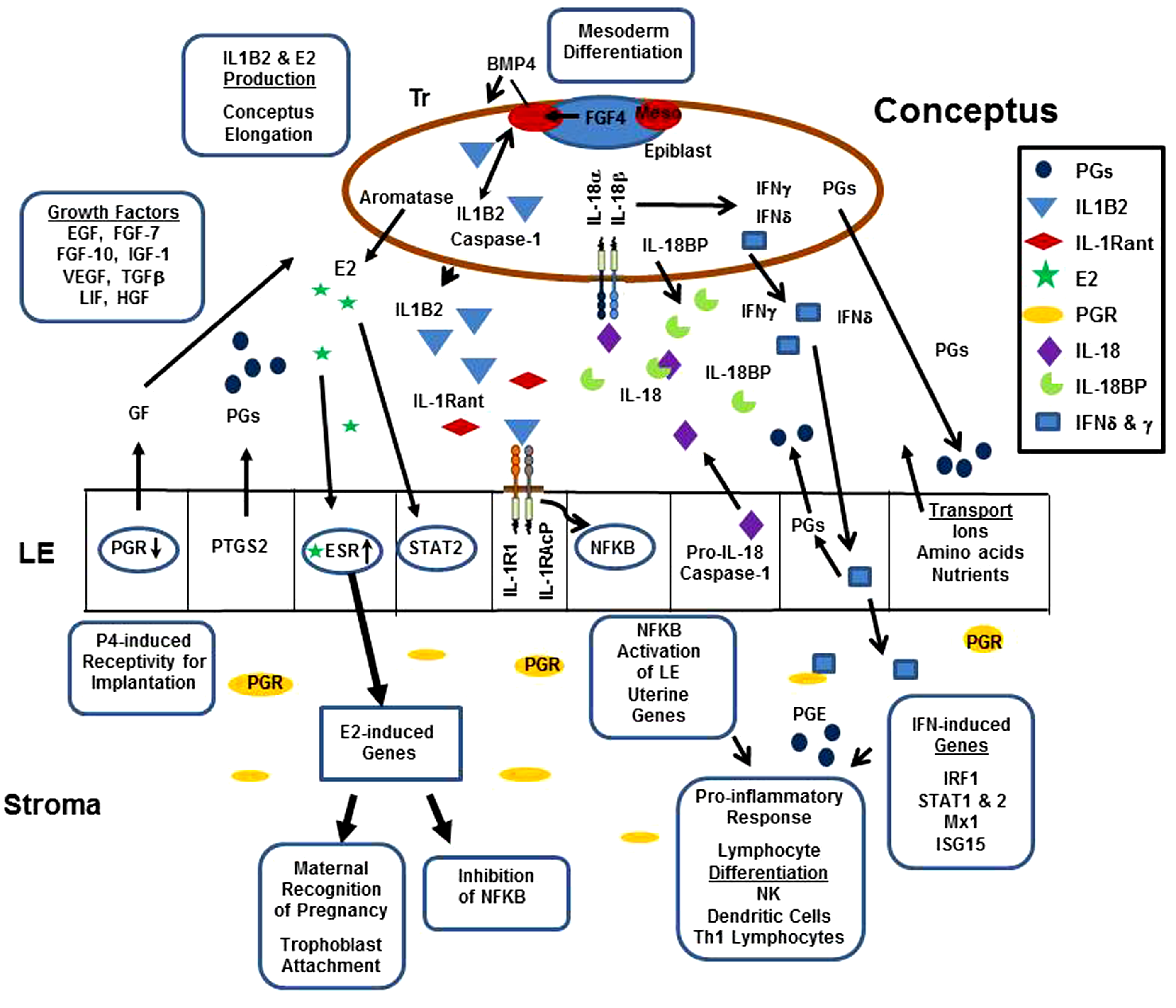

Supplement: Supplementary file 3 — Authors’ original file for figure 3 [file 40104_2014_129_MOESM3_ESM.tif]
